# The impact of switching to mTOR inhibitor-based immunosuppression on long-term non-melanoma skin cancer incidence and renal function in kidney and liver transplant recipients

**DOI:** 10.1080/0886022X.2020.1785499

**Published:** 2020-07-01

**Authors:** Susan L. Murray, Fergus E. Daly, Patrick O’Kelly, Eamonn O’Leary, Sandra Deady, James P. O’Neill, Alexander Dudley, Nicholas R. Rutledge, Aiden McCormick, Diarmuid D. Houlihan, Yvonne Williams, Patrick G. Morris, Siona Ni Raghallaigh, Fergal J. Moloney, Donal J. Sexton, Peter J. Conlon

**Affiliations:** a Department of Nephrology & Transplantation, Beaumont Hospital, Dublin, Ireland; b Department of Medicine, Royal College of Surgeons in Ireland, Dublin, Ireland; c National Cancer Registry Ireland, Cork Airport Business Park, Cork, Ireland; d Department of Otolaryngology, Head & Neck Surgery, Beaumont Hospital, and Royal College of Surgeons, Ireland, Ireland; e Hepatology & Liver Transplant Department, St Vincent’s University Hospital, Dublin, Ireland; f Department of Oncology, Beaumont Hospital, Dublin, Ireland; g Department of Dermatology, Beaumont Hospital, Dublin, Ireland; h Department of Dermatology, Mater Misericordia University Hospital University College, Dublin, Ireland

**Keywords:** Immunosuppression, cancer, skin cancer, mTOR inhibitor, renal transplant, liver transplant

## Abstract

**Background:**

Solid organ transplantation is associated with increased risk of non-melanoma skin cancer. Studies with short follow up times have suggested a reduced occurrence of these cancers in recipients treated with mammalian target of rapamycin inhibitors as maintenance immunosuppression. We aimed to describe the occurrence of skin cancers in renal and liver transplant recipients switched from calcineurin inhibitor to sirolimus-based regimes.

**Methods:**

We performed a retrospective study of sirolimus conversion within the Irish national kidney and liver transplant programs. These data were linked with the National Cancer Registry Ireland to determine the incidence of NMSC among these recipients. The incidence rate ratio (IRR) for post versus pre-conversion NMSC rates are referred in this study as an effect size with [95% confidence interval].

**Results:**

Of 4,536 kidney transplants and 574 liver transplants functioning on the 1 January 1994 or transplanted between 1 January 1994 and 01 January 1994 and 01 January 2015, 85 kidney and 88 liver transplant recipients were transitioned to sirolimus-based immunosuppression. In renal transplants, the rate of NMSC was 131 per 1000 patient years pre-switch to sirolimus, and 68 per 1000 patient years post switch, with adjusted effect size of 0.48 [0.31 − 0.74] (*p* = .001) following the switch. For liver transplant recipients, the rate of NMSC was 64 per 1,000 patient years pre-switch and 30 per 1,000 patient years post switch, with an adjusted effect size of 0.49 [0.22 − 1.09] (*p* .081). Kidney transplant recipients were followed up for a median 3.4 years. Liver transplants were followed for a median 6.6 years.

**Conclusions:**

In this study, the conversion of maintenance immunosuppression from calcineurin inhibitors to mTOR inhibitors for clinical indications did appear to reduce the incidence of NMSC in kidney and liver transplant recipients.

## Introduction

Renal transplantation is the treatment of choice for end stage renal disease (ESRD), imparting longer survival and better quality of life [1]. However, there are a number of complications associated with the required immunosuppression, including a significant increase in the incidence of cancer, which represents a significant risk of morbidity and mortality in transplant recipients [2]. Much of the increased cancer incidence is driven by the rate of the non-melanoma skin cancers (NMSC), including squamous cell carcinoma (SCC) and basal cell carcinoma (BCC) that occur in up to 50% of all Caucasian, solid organ transplant recipients [3–5] Transplant recipients are up to 100 times more likely to develop NMSC, and of those that do, 30–70% will go on to develop subsequent NMSC [6,7]. Skin cancer, and particularly SCC, tends to be more aggressive in solid organ recipients, with more primaries, deeper tissue spread and more lymphatic or perineural invasion [8].

While immunosuppression burden is associated with NMSC development in general, some immunosuppressants may promote this development more than others [9]. Conversely, treatment with mammalian target of rapamycin (mTOR) inhibitors, such as sirolimus, has been shown to reduce the incidence of cancer [10,11]. The CONVERT trial found a reduced incidence of malignancy amongst renal transplant recipients over two years of follow up with a conversion from calcineurin inhibitors to sirolimus-based immunosuppression [12]. This treatment effect has been reproduced in other randomized controlled trials [13,14]. Meta-analysis of the effect of calcineurin inhibitors did not, however, reach significance and showed only a trend toward cancer reduction [15,16]. A retrospective study of solid organ recipients, including liver transplant recipients, also showed a decreased risk of NMSC [17].

There is little evidence available to show if converting to mTOR inhibitors results in reduced skin cancer incidence over longer term follow up outside of the randomized trial context. This study was undertaken to determine whether the number of NMSC decreases over time, following a switch to sirolimus, in a consecutive sample of kidney and liver transplant recipients.

## Materials & methods

This was a retrospective study of sirolimus conversion within the Irish National Kidney Transplant program and the Irish National Liver Transplant Program. Ethical approval was obtained (CA95). Electronic health records were reviewed to identify renal and liver transplant recipients who were commenced on sirolimus in clinical practice. The Irish National Kidney Transplant program has performed more than 5,000 kidney transplants, while the Irish National Liver Transplant Program has performed over 600 liver transplants. The programs have 18 years’ experience of mTOR inhibitor-based immunosuppression.

The decision to switch recipients from calcineurin-based immunosuppression was based on the individual clinician’s clinical judgment. These data were linked with the National Cancer Registry Ireland (NCRI) to determine the incidence of NMSC amongst these recipients. The NCRI prospectively gathers data on all cancers in Ireland. It has been collecting such data since 1994. Their case identification relies primarily on reports from pathology departments, with further cases registered from sources including electronic coding records, reports from oncology and radiology departments, medical charts, and hospital cancer databases. Data gathered include demographics, the type and location of cancer, and the degree of invasiveness.

We reviewed all recipients who were transplanted after 01 January 1994, the date when the NCRI began collecting data, or who had a functioning transplant on that date. Recipients whose immunosuppression had been converted to sirolimus at any point from the date of kidney or liver transplantation, until the 1st of January 2015 were included in this study. Recipients who were switched due to a malignancy other than NMSC and those whose initial immunosuppression on transplantation included sirolimus were excluded. Recipients were not censored for duration of switch to sirolimus. ICD10 coding was used to determine cancer diagnosis. ICD10 Codes C44.0–C44.9 & D04 for SCC, BCC and SCC *in situ* were included.

We measured serum creatinine concentration one year before and one year switch to sirolimus to assess for any change in renal function after switch to sirolimus.

## Statistical methods

Poisson regression models were used for the primary analysis where count data were transformed to incidence rates based on patient exposure time. Incidence rate ratios (IRR) were derived for the main switching effect along with confounding variables of age and sex. For the purpose of this study we define IRR in the more generalized term of effect size. Effect sizes were calculated for the complete follow up times for each individual patient and for one year pre and post switch. Mann Whitney tests were used to compare renal function before and after immunosuppression switch. Frequency of NMSC over time (which incorporated switch time = 0) were presented on kernel density function graphs.

Statistical analyses were performed using Stata 13 SE (College Station, Texas). Results were deemed to be significant at the 5% level.

## Results

### Renal transplant recipients

A total of 4,536 renal transplants had a functioning transplant on 01 January 1994 or were transplanted between 01 January 1994 and 01 January 2015 in the Republic of Ireland. Of these, 85 renal transplants (1.9%) transitioned to sirolimus at some point in their post-transplant course. The number of male recipients was 63 (74%) The median age at transplant was 44 years (Range: 10–77). (Table 1). The most common reason for recipients to switch to sirolimus was calcineurin toxicity, or fibrosis, in 46 recipients (54.2%), while one or more previous malignancies accounted for the decision to change in the other 39 recipients (45.8%).

**Table 1.** Characteristics of liver and kidney transplant recipients switched to sirolimus maintenance immunosuppression.

	Kidney Transplant	Liver Transplant
	(*n* = 85)	(*n* = 88)
Median age at transplant	44 (10–77)	55 (21–70)
Median age at switch to sirolimus	51 (11–80)	58 (22–73)
Male Sex	63 (74%)	63 (72%)
Caucasian Ethnicity	85 (100%)	87 (99%)
Indication for Switch		
Malignancy	39 (45.8%)	10 (11.4%)
Calcineurin Toxicity	46 (54.2%)	51(58.0%)
Other	0 (0%)	27 (30.6%)
Immunosuppressive Regime Pre-Switch		
Tacrolimus & Mycophenolate	28 (33%)	57 (65%)
Tacrolimus Alone	0 (0%)	21 (24%)
Ciclosporin & Mycophenolate	3 (3.5%)	2 (2%)
Tacrolimus & Azathioprine	8 (9%)	5 (6%)
Ciclosporin & Azathioprine	37 (44%)	1 (1%)
Azathioprine Alone	3 (3.5%)	0 (0%)
Ciclosporin Alone	5 (6%)	0 (0%)
Mycophenolate alone	0 (0%)	2 (2%)
Unknown	1 (1%)	0 (0%)
Immunosuppression Regime Post-Switch		
Sirolimus Alone	18 (21%)	25 (28%)
Sirolimus & Azathioprine	16 (19%)	1 (1%)
Sirolimus & Mycophenolate	51 (60%)	50 (57%)
Sirolimus, Tacrolimus & Mycophenolate	0 (0%)	4 (5%)
Sirolimus & Tacrolimus	0 (0%)	8 (9%)

The median time from transplantation to switch was 2,791 days (95% CI: 2,236–3,347) or 7.6 years. Recipients were followed for a median 3.4 years following switch from calcineurin inhibitor to sirolimus.

Recipients were on a variety of immunosuppressive regimes before switching to sirolimus. All recipients were on some combination of azathioprine, tacrolimus, ciclosporin and corticosteroids. Before switching to sirolimus, the most common immune regime was ciclosporin and azathioprine (44%), followed by tacrolimus and mycophenolate (33%). After the switch to sirolimus, the most common maintenance immunosuppressant regime was a combination of sirolimus and mycophenolate, with 51 recipients (60%) taking this combination, 19% took azathioprine and sirolimus and 21% were on sirolimus alone.

Prior to the switch to sirolimus, 42 recipients had all developed at least one NMSC or NMSC-in-situ, 26 had developed two or more, and three had developed more than five. The greatest number of cancers an individual had developed prior to the switch to sirolimus was nine.

**Figure 1.**
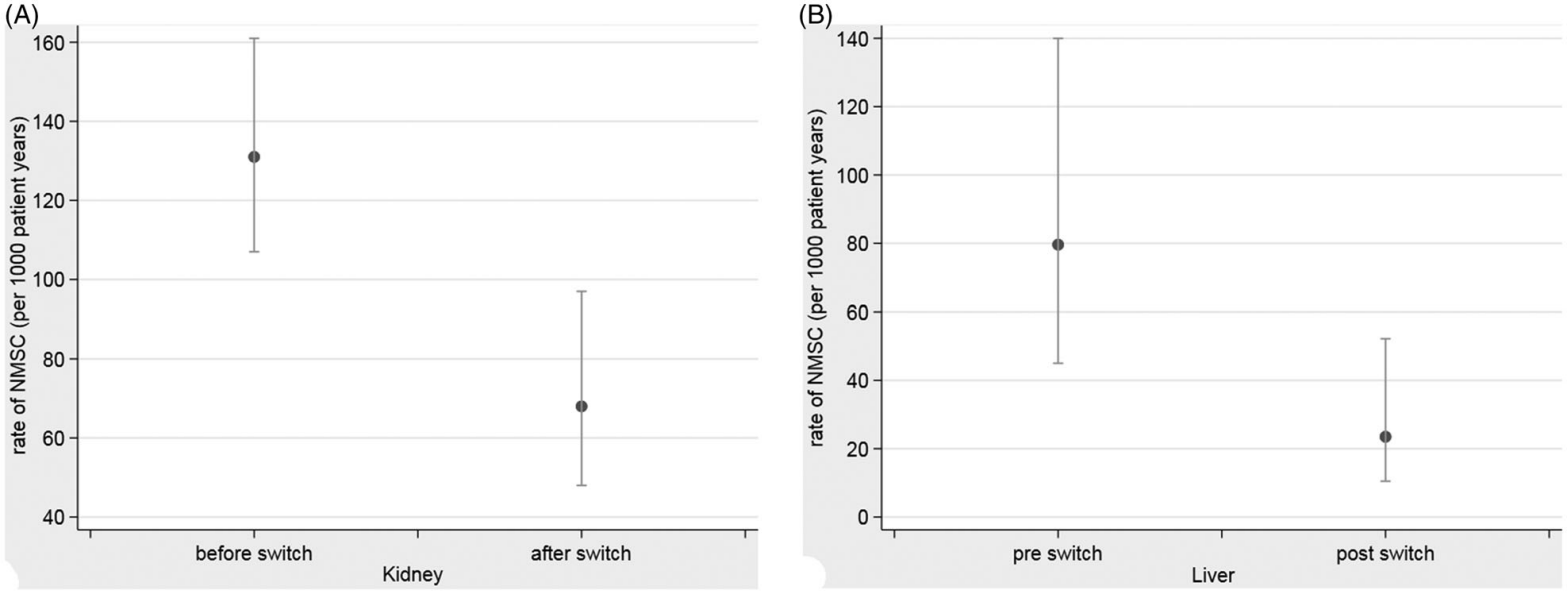
(A) Rate of NMSCs in renal transplant recipients before and after switch to sirolimus. (B) Rate of NMSCs in liver transplant recipients before and after switch to sirolimus.

The rate of development of NMSC pre-switch to sirolimus was 131 per 1000 patient years. After the switch to sirolimus, those that switched went on to develop a total of 30 further skin cancers. Only four recipients (4.7%) developed more than one other skin cancer post-switch to sirolimus. The maximum number of skin cancers that developed post switch was three. The rate of NMSC after the switch was 68 NMSC per 1000 patient years. (See Figure 1(A)).

For all pre and post follow up times, the age and sex adjusted effect size of skin cancer following the switch to sirolimus was 0.48 [0.31 − 0.74] (*p* = .001).

**Figure 2.**
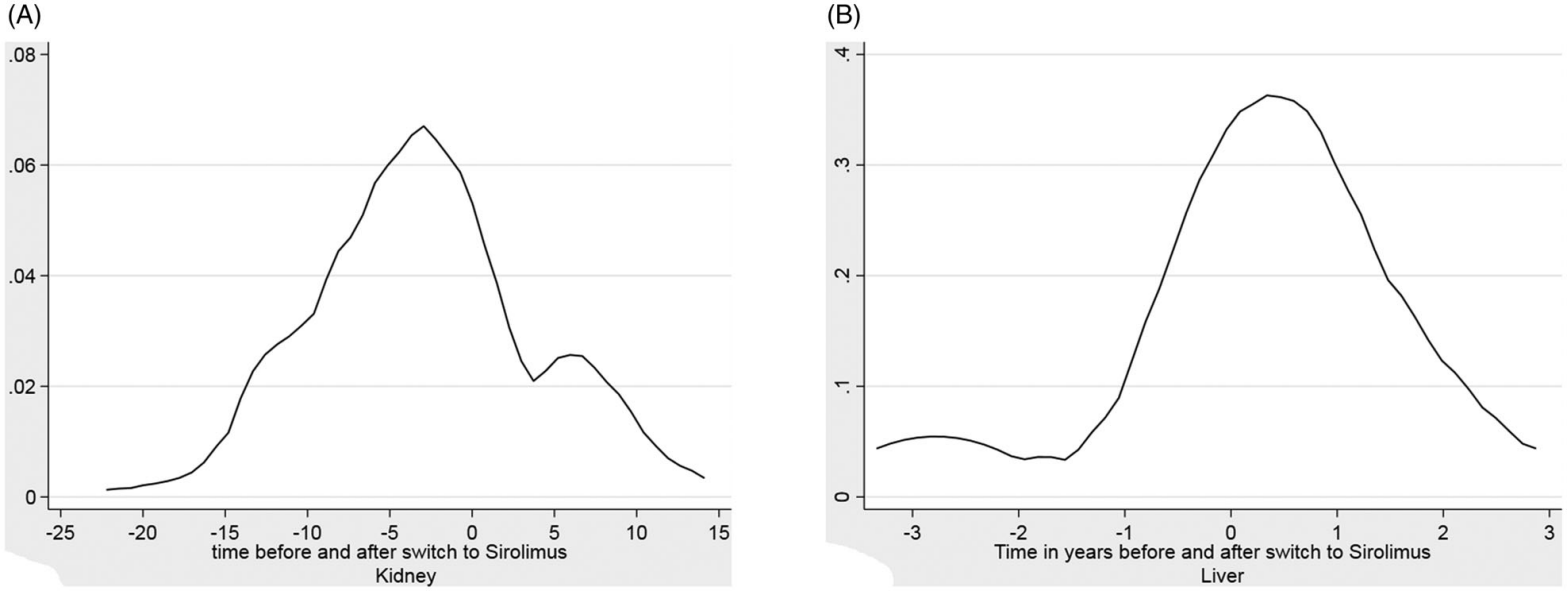
(A) Kernel Density plots of NMSC in renal transplant recipients before nd after switch to sirolimus. (2) Kernel density of NMSC in liver transplant recipients before and after switch to sirolimus.

For one year pre and post switch the adjusted effect size of skin cancer following the switch to sirolimus was 0.17 [0.05 − 0.61] (*p* = .007). Figure 2(A) describes the distribution of the rate of NMSC diagnosis in renal transplants, punctuated by the switch to sirolimus.

**Figure 3.**
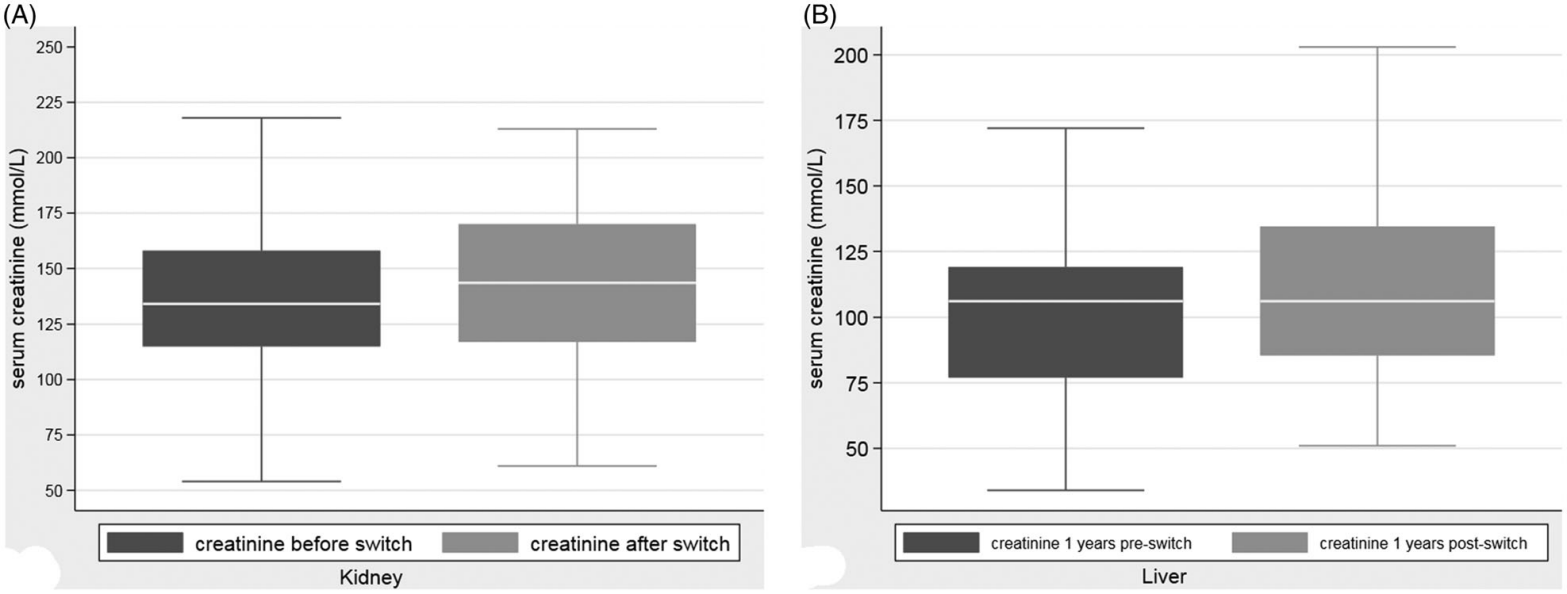
(A) Serum creatinine concentration (umol/L) one year before and one year after switch to sirolimus in renal transplants. (B) Serum creatinine concentration one year before and one year after switch to sirolimus in liver transplants.

The switch from alternative immunosuppressive regimes to sirolimus did not have a significant impact on renal function (Figure 3(A)). Median creatinine before the switch was 135 μmol/L, after the switch it increased to 144 μmol/L.

### Liver transplant recipients

A total of 574 liver transplant recipients had a functioning transplant on 01 January 1994 or were transplanted between 01 January 1994 and 01 January 2015 in the Republic of Ireland. Overall, 88 liver transplants (15.3%) transitioned to sirolimus at some time in their post-transplant course. Among those 88 recipients, the number of male recipients was 63 (72%) and 87 recipients (99%) were Caucasian. The median age at transplant was 55 years (Range: 21–70). (Table 1). The primary indication for switch from calcineurin-based immunosuppression to sirolimus-based immunosuppression was for renal impairment attributed to calcineurin nephrotoxicity in 51 recipients, (58%), while 10 recipients (11.4%) were switched because of previous malignancies.

The median time from transplantatiom to switch to sirolimus was 1,091 days (95% CI: 758–1,424) or three years. Recipients were followed for a median 6.6 years following switch from calcineurin inhibitor to sirolimus.

Before switching to sirolimus, the most common immune regime was tacrolimus and mycophenolate (65%), followed by tacrolimus alone (24%). After the switch to sirolimus the most common regime was a combination of sirolimus and mycophenolate (57%), while 28% took sirolimus only.

Prior to switching to sirolimus, nine liver transplant recipients (10.2%) had developed NMSC and three (3.4%) had developed multiple skin cancers. No recipient had developed more than two NMSCs. The rate of NMSC pre-switch to sirolimus was 64 per 1000 patient years. After switching to sirolimus, twelve liver transplant recipients (14%) who had switched developed a further NMSC. No liver transplant recipient developed more than one skin cancer post switch.

The rate of NMSC after the switch was 30 per 1000 patient years (Figure 1(B)). The adjusted effect size for all pre and post switch times for liver transplant recipients was 0.49 [0.22 − 1.09] (*p* = .081).

For one year pre and post switch the adjusted effect size of skin cancer following the switch to sirolimus was 0.45 [0.11 − 1.89] (*p* = .276).

Figure 2(B) describes the distribution of the rate of NMSC diagnosis in liver transplants punctuated by the switch to sirolimus.

There was a non-significant change in serum creatinine concentration from one-year pre-transplant to one-year post-transplant of 108–109 μmol/L (Figure 3(B)), an overall change of 1 μmol/L

## Discussion

In this study describing national registry data of kidney and liver transplant recipients transitioned from calcineurin to sirolimus-based immunosuppression, we observed a marked reduction in the occurrence of NMSC over follow up, without a consequent deterioration in renal function.

Our study followed 173 solid organ transplant recipients who switched from calcineurin-based to sirolimus based-immunosuppression. This is one of the largest observational studies of conversion to sirolimus after transplant, with comparable cohort size to previous studies and a median follow up nearly twice that of previous studies [18,19].

Recipients switched for a variety of reasons, but predominantly either to attempt to reduce the risk of further cancers in patients who had developed previous NMSC or due to nephrotoxicity attributed to calcineurin inhibitors. Our population was almost entirely Caucasian, drawn from a population with predominantly fair, Fitzpatrick type I & type II skin, the skin types at highest risk for skin cancer [20]. Following conversion to sirolimus there were 50% less individual NMSCs across all recipients in both transplant groups, with a median of 3.4 years follow up in renal transplant recipients. This is in line with previously reported rates in randomized controlled trials [12,13].

The NMSC rate reduction compared to those on a calcineurin-based regime may be related to calcineurin inhibitor withdrawal, as they are thought to affect surveillance mechanisms on cells undergoing neoplastic differentiation, prevent DNA repair mechanisms and promote tumor growth [21]. However, equally, mTOR inhibitors are thought to have anti-proliferative effects which may reduce the risk of cancer. This may be attributable to inactivation of the mTOR protein kinase, which regulates the cell cycle and is needed to drive tumorigenesis once an oncogene has been activated [10,22]. The angiogenesis required for tumor growth is inhibited by mTOR inhibitor use, leading to a slowing of the proliferation of malignant cells.

Our results align with a recent meta-analysis suggesting there was a decreased risk of malignancy in those with kidney transplants on a sirolimus-based regime [16]. This meta-analysis, with a median follow up of four years, did show that the risk of death was higher in those receiving the sirolimus regime, which may be related to indication bias related to the switch.

Sirolimus appears to be a good alternative for preservation of renal function post-transplant. The switch to sirolimus from tacrolimus in our study had no demonstrable adverse effect on renal function, since no significant difference was observed between those on the sirolimus and non-sirolimus-based regimes. This is supported by the results in the CONVERT trial, which showed a higher GFR in those on sirolimus who had a GFR >40 mL/min/1.73m^2^ [12].

Our analysis does not allow us to identify who would most benefit from the switch to mTOR inhibitor or if all those who develop a single NMSC should be switched to MTOR inhibitors. Further prospective analysis would need to be carried out to assess this.

In recent years, there has been an increasing appreciation of the effects of chronic immune-mediated rejection on graft loss. As such, calcineurin inhibitor-sparing strategies to combat chronic allograft nephropathy are being pursued less frequently. However, our study suggests there may still be a role for its use in those who develop multiple skin cancers. Augmentation to an mTOR inhibitor-based immunosuppression regime was associated with reduced occurrence of NMSC in both liver and kidney transplant recipients in this study.
